# (Epi) Genetics and the complexity of diabetes mellitus

**DOI:** 10.20945/2359-3997000000002

**Published:** 2018-01-01

**Affiliations:** 1 Universidade de São Paulo Universidade de Sao Paulo Faculdade de Medicina Laboratório de Carboidrato e Radioimunoensaio (LIM-18), Hospital das Clinicas Sao Paulo SP Brasil Laboratório de Carboidrato e Radioimunoensaio (LIM-18), Hospital das Clinicas, Faculdade de Medicina, Universidade de Sao Paulo (HCFMUSP), Sao Paulo, SP, Brasil. Programa de Pós-Graduação em Medicina, Universidade Nove de Julho, Sao Paulo, SP, Brasil; Universidade Nove de Julho Universidade Nove de Julho Sao Paulo SP Brasil

Both type 1 (T1D) and type 2 (T2D) diabetes mellitus are complex diseases to which environmental and genetic factors contribute (1). Recently, epigenetic factors have also been recognized as important not only in the etiopathogenesis of these conditions, but also in the development of their chronic complications (2). As if the interaction among environment, genetics and epigenetics is not complex enough, each of these factors has their own complexity. For instance, regarding the genetic setting, both T1D and T2D are polygenic diseases for which several susceptibility genes contribute, each with a relatively small participation (1). In addition, susceptibility genes may vary among different populations, reason why genetic studies should be reproduced in the population of interest.

In this issue of *Archives of Endocrinology and Metabolism* (AE&M), two manuscripts address, respectively, genetic and epigenetic factors associated with diabetes. Pirozzi and cols. (3) evaluated in a population of Brazilian obese patients from the Southeast region, the association of T2D with two polymorphisms, rs1799752 in the gene encoding angiotensin I converting enzyme (*ACE*) and rs1801133 in the gene encoding methylenetetrahydrofolate reductase (*MTHRF*). MTHRF is the enzyme that catalyzes the conversion of 5,10-methylenetetrahydrofolate to 5-methyltetrahydrofolate whose deficiency increases plasmatic homocysteine concentrations. Both polymorphisms had been previously studied for the association to T2D with conflicting results in different ethnic groups (4,5). No association of these polymorphisms with T2D was found by Pirozzi and cols., in agreement with a previous study performed in Brazilian patients from the South region, which also did not find association of T2D with rs1799752 in the *ACE* gene (6). Given the admixture which characterizes the Brazilian population and the distinct genetic background even among the different Brazilian regions (7), it is important that such genetic studies are carried out, preferably in larger series of patients.

Micro RNAs (miRNAs) are one of the epigenetic mechanisms; they are small non-coding RNAs that repress gene expression at the post transcriptional level (8) and because they regulate several cellular processes, they have been implicated in human diseases (9). miRNA expression profiles have been explored as a potential tool to classify disease states and, in certain situations, to diagnostic applications (10). In the second manuscript, García-Días and cols. evaluated the expression of three miRNAs in peripheral blood mononuclear cells (PBMC) from T1D patients as compared to control (non-diabetes) subjects. A higher expression of miR-155 and a lower expression of miR-326 and miR-146a were observed in T1D patients in comparison to non-diabetes subjects. Interestingly, miR-155 expression was associated with autoimmunity and with the inflammatory status (11). It had been previously demonstrated that different inflammatory mediators, such as tumor necrosis factor (TNF), are able to induce miR-155 expression in macrophages (12). Because hyperglycemia increases the production of TNF and other cytokines, it is possible that increased miR-155 expression in PBMC from T1D patients reflects a sustained inflammatory state associated with the suboptimal metabolic control (11). Studies like this one exploring the association between clinical and biochemical variables and aberrant expression of miRNAs can improve the understanding of the epigenetic mechanisms triggered by longstanding hyperglycemia, which underlie the concept of Metabolic memory.
